# SLAMF7 modulates B cells and adaptive immunity to regulate susceptibility to CNS autoimmunity

**DOI:** 10.1186/s12974-022-02594-9

**Published:** 2022-10-03

**Authors:** Patrick O’Connell, Maja K. Blake, Sarah Godbehere, Andrea Amalfitano, Yasser A. Aldhamen

**Affiliations:** 1 Department of Microbiology and Molecular Genetics, College of Osteopathic Medicine, Michigan State University, 567 Wilson Road, 4108 Biomedical and Physical Sciences Building, East Lansing, MI 48824 USA; 2 Department of Pediatrics, College of Osteopathic Medicine, Michigan State University, East Lansing, MI 48824 USA

**Keywords:** SLAMF7, CD319, CRACC, Multiple sclerosis, B cells, Neuroinflammation, T cells, SLAMF9, Disease-associated microglia, EAE

## Abstract

**Background:**

Multiple sclerosis (MS) is a chronic, debilitating condition characterized by CNS autoimmunity stemming from a complex etiology involving both environmental and genetic factors. Our current understanding of MS points to dysregulation of the immune system as the pathogenic culprit, however, it remains unknown as to how the many genes associated with increased susceptibility to MS are involved. One such gene linked to MS susceptibility and known to regulate immune function is the self-ligand immune cell receptor SLAMF7.

**Methods:**

We subjected WT and SLAMF7^−/−^ mice to multiple EAE models, compared disease severity, and comprehensively profiled the CNS immune landscape of these mice. We identified all SLAMF7-expressing CNS immune cells and compared the entire CNS immune niche between genotypes. We performed deep phenotyping and in vitro functional studies of B and T cells via spectral cytometry and BioPlex assays. Adoptive transfer studies involving the transfer of WT and SLAMF7^−/−^ B cells into B cell-deficient mice (μMT) were also performed. Finally, B–T cell co-culture studies were performed, and a comparative cell–cell interaction network derived from scRNA-seq data of SLAMF7^+^ vs. SLAMF7^−^ human CSF immune cells was constructed.

**Results:**

We found SLAMF7^−/−^ mice to be more susceptible to EAE compared to WT mice and found SLAMF7 to be expressed on numerous CNS immune cell subsets. Absence of SLAMF7 did not grossly alter the CNS immune landscape, but allowed for altered immune cell subset infiltration during EAE in a model-dependent manner. Global lack of SLAMF7 expression increased myeloid cell activation states along with augmented T cell anti-MOG immunity. B cell profiling studies revealed increased activation states of specific plasma and B cell subsets in SLAMF7^−/−^ mice during EAE, and functional co-culture studies determined that SLAMF7^−/−^ B cells induce exaggerated T cell activation. Adoptive transfer studies revealed that the increased susceptibility of SLAMF7^−/−^ mice to EAE is partly B cell dependent and reconstruction of the human CSF SLAMF7-interactome found B cells to be critical to cell–cell communication between SLAMF7-expressing cells.

**Conclusions:**

Our studies have identified novel roles for SLAMF7 in CNS immune regulation and B cell function, and illuminate underpinnings of the genetic association between SLAMF7 and MS.

**Supplementary Information:**

The online version contains supplementary material available at 10.1186/s12974-022-02594-9.

## Introduction

Autoimmune diseases represent some of the most pathologically complex conditions in modern medicine, both in terms of their causes and response to therapies. Multiple sclerosis (MS) is no exception, and takes a significant toll on those it affects as it often appears early in life, progressively gets worse, and consequently, leads to many years of disability and hardship [1]. Since MS was first termed in 1868 [2], efforts have been underway to identify both the underlying causative mechanisms and effective treatments. Our current understanding of MS has identified both environmental and genetic linkages to MS susceptibility [3], with immune dysfunction emerging as a unifying feature [4]. However, the immune system is recognized as one of the human body’s most complex organ systems, and we still have much to learn regarding how complex immune interaction networks maintain health and drive disease susceptibility [5].

While there are a considerable number of available therapies for MS, most of them targeting the immune system [6–8], many patients, particularly those with more aggressive forms of the disease, fail to respond to these therapies [9]. An example is the newer B cell-depleting antibodies (ocrelizumab, ofatumumab, etc.) which have shown remarkable success in inducing remissions in MS patients with the relapsing remitting subtype [8, 10], yet are less effective in patients with the more severe primary progressive subtype [9]. To increase our understanding about immune cell dysregulation in MS, we have undertaken efforts to understand how a specific gene linked to MS contributes to neuroinflammation and disease pathogenesis.

The International Multiple Sclerosis Genetics Consortium has made great strides in identifying the plethora of genes linked to MS susceptibility [4], yet few of these genes have been investigated in-depth to determine mechanistically how they contribute to susceptibility to CNS autoimmunity. Here, we investigated the link between one such gene previously associated with MS, the signaling lymphocytic activation molecule (SLAM) family member 7 (*SLAMF7*) gene [4, 11, 12]. The largest genome-wide association study performed to date involving tens of thousands of MS patients and controls identified the rs983494 SNP, located in the promoter of *SLAMF7*, as being significantly linked to MS susceptibility [4]. While this SNP does not lie in a *SLAMF7* exon, cis-eQTL studies of this SNP with peripheral blood mononuclear cells (PBMCs) revealed a significant cis-eQTL with *SLAMF7* expression [4] and single-tissue eQTLs show this SNP significantly decreases SLAMF7 expression (GTExPortal), suggesting this SNP can alter SLAMF7 expression. SLAMF7 is expressed only on hematopoietic cells and can drive either activating or inhibitory functions in immune cells, a mechanism that is dependent on the presence or absence of its intracellular adaptor protein, Ewing sarcoma-associated transcript 2 (EAT-2) [13, 14]. In general, in cells that express EAT-2, SLAMF7 signaling is activating and in the absence of EAT-2, it is inhibitory [13, 15]. We have previously defined roles for SLAMF7 in a number of conditions including HIV infection [15] and cancer [16], and have also worked to modulate SLAMF7 signaling for therapeutic benefit [17–23].

To define the role of SLAMF7 in CNS inflammation and MS pathogenesis, we used the murine model of MS, experimental autoimmune encephalomyelitis (EAE), and found SLAMF7^−/−^ mice to be more susceptible to CNS autoimmunity compared to their WT counterparts. We also characterized SLAMF7 expression across the CNS immune landscape. We further defined CNS immune cell-specific changes driven by SLAMF7 and identified a role for SLAMF7 in the modulation of B cell and T cell memory responses. Deep phenotyping and functional studies highlighted specifically how SLAMF7 alters B and plasma cell responses, while adoptive transfer studies confirmed that SLAMF7 signaling on B cells regulates T cell responses and modulates EAE susceptibility. Finally, a targeted re-analysis of *SLAMF7-*expressing CSF immune cells from scRNA-seq data in MS patients and healthy controls identifies *SLAMF7*^+^ B cells as a cellular hub for cell–cell interactions between *SLAMF7-*expressing immune cells. These studies identified a novel role for SLAMF7 in the modulation of B cell and memory T cell responses, and provided early mechanistic evidence supporting the genetic link between *SLAMF7* and MS.

## Materials and methods

### EAE model

**Fig. 1:**
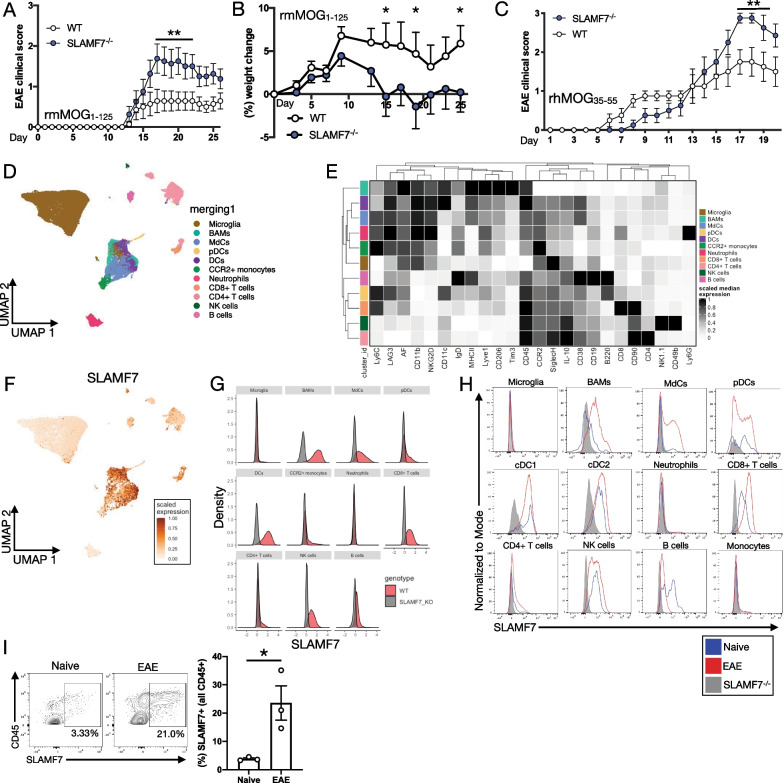
SLAMF7 displays unique expression patterns in the CNS and protects from EAE. **A** Clinical scores of WT (*n* = 14) and SLAMF7^−/−^ mice (*n* = 16) subjected to EAE induced with rmMOG_1–125_. **B** Change in baseline weight for mice in **A**. **C** Clinical scores of WT (*n* = 8) and SLAMF7^−/−^ mice (*n* = 8) subjected to EAE induced with rhMOG_35–55_. **D** UMAP of the entire CNS immune landscape in WT and SLAMF7^−/−^ mice subjected to EAE with rmMOG_1–125_. The complete CNS immune landscape was assessed via high-dimensional single-cell spectral cytometry. **E** Marker expression on CNS immune cell subsets from **D**. **F** Scaled SLAMF7 expression plotted on the UMAP projection of all CNS immune cells from **D**. **G** Density plots of SLAMF7 expression across CNS immune subsets from **D**. **H** Histograms of SLAMF7 expression on CNS immune cell subsets at steady state and during active EAE (with rmMOG_1–125_) neuroinflammation. **I** SLAMF7 expression on all CNS immune cells at steady state and during EAE (with rmMOG_1–125_). Groups in **A** compared with a two-way ANOVA with Sidak’s multiple comparison test, displayed with mean ± SEM, and representative of two independent experiments showing similar results. Groups in **B** compared with a two-way ANOVA with Fisher’s LSD test. Groups in **C** compared with a mixed-effects model with Sidak’s multiple comparison test, displayed with mean ± SEM, and representative of a single experiment. Groups in **I** compared with an unpaired two-way *t*-test and representative of two independent experiments showing similar results. *p < 0.05, **p < 0.01. BAMS, border-associated macrophages; MdCs, myeloid-derived cells; AF, autofluorescence

SLAMF7^−/−^ mice were generated as previously described [16]. For the rmMOG_1–125_ model, mice were injected on day −2 with 33 μg full-length recombinant murine MOG (rmMOG_1–125_) protein (Anaspec) with equal parts complete Freund’s adjuvant (CFA) as previously described [24]. 400 ng of pertussis toxin (MilliporeSigma) was injected intraperitoneally on days −2 and 0. For the rhMOG_35–55_ model, EAE was induced as described above, except 300 µg of rhMOG_35–55_ peptide was used. Mice were scored daily on a scale of 0–5 as previously described [24], with a score of 0 = no symptoms, 1 = tail paresis, 2 = partial hindlimb paresis, 3 = complete hindlimb paresis, 4 = complete hindlimb paresis and front limb involvement, 5 = moribund or dead. Mice were humanely euthanized if a score of 4 or 5 was reached. Investigators were blinded to genotypes of mice during the first experiment comparing WT and SLAMF7^−/−^ mice (presented in Fig. 1). The WT mice used in experiments depicted in Fig. 1A, B were from a combined experiment, the remainder of which is available elsewhere [25].

### Adoptive transfer study

Splenocytes were collected from WT and SLAMF7^−/−^ mice as previously described [26]. B cells were isolated per manufacturer’s guidelines using the murine B cell isolation kit (Miltenyi Biotec) and injected I.V. retro-orbitally (2 × 10^7^cells) into μMT mice (The Jackson Laboratory). Five days after transfer, EAE was induced in mice with rmMOG_1–125_ protein as described above.

### CNS immune cell isolation

All CNS and splenic immune cell isolations, including in vivo labeling and anti-CD45 were performed precisely as previously described [25].

### Spectral cytometry

Cells were isolated from the CNS and spleen as previously described [21]. Cells were stained with various antibodies (Additional file 2: Table S1) utilizing BD Brilliant Stain Buffer (50 uL per sample) whenever multiple Brilliant dyes were used in combination. Viability staining was performed with Zombie NIR (BioLegend) and Fc receptors were blocked with murine Fc block (BD Biosciences). Samples were acquired on a 5 laser Cytek Aurora Spectral Cytometer and data were analyzed using FlowJo version 10.6.1 (Tree Star) and the R computing environment. High-dimensional single-cell spectral cytometry was performed in R. The CATALYST package was used to perform all analyses with a cell annotation and dimensionality reduction approach as previously described [23, 25, 27]. Data clean up and further analyses were performed as previously described [25].

For experiments using IL-10^GFP^ mice and/or in vivo labeling analysis was performed as previously described [25].

### Confocal microscopy

EAE was induced in WT mice as described above and during the peak of EAE severity, mice were killed and brain tissue was harvested. Mice were perfused with 10% formalin before collection of brain tissue which was stored in 10% formalin for approximately 24 h before being transferred into a 30% sucrose solution for approximately 72 h. Brains were sectioned at 4 μm on a cryotome. Tissue was blocked with 3% normal donkey serum and 0.3% Triton X for 40 min at 4 °C. Primary staining with anti-SLAMF7 (1:500) and anti-Iba1 (1:1000) was performed overnight at 4 °C in buffer containing 3% normal donkey serum and 0.3% Tween-20. Sections were washed with PBST and PBS before secondary staining with Alexa 555 anti-rabbit (1:1000) and Alexa 488 anti-goat (1:1000) at room temperature for 5.5 h. Sections were rinsed in PBS, mounted on slides, and counter stained with DAPI using ProLong Gold antifade reagent (Invitrogen). Sections were imaged with an Olympus FluoView 1000 CLSM.

### Multiplex cytokine/chemokine analysis

Mouse 23-analyte multiplex-based assay was used to determine cytokine/chemokine concentrations via Luminex 100 per manufacturer’s protocol (Bio-Rad), as previously described using a 1:4 dilution of plasma [15, 18].

### B cell:T cell co-culture study

Pan-B cells were isolated from WT and SLAMF7^−/−^ mice per manufacturer’s protocol (Miltenyi Biotec). B cells were labeled with CFSE (ThermoFisher) per manufacturer guidelines and pulsed with MOG_1–125_ protein (10 μg/mL) overnight. Cells were cultured with IL-2 (5 ng/mL) as well. The next day, splenocytes were isolated from WT mice previously challenged with the EAE model, CD3^+^ T cells were isolated per manufacturer protocol (Miltenyi Biotec), and labeled with CellTrace Violet (ThermoFisher) per manufacturer’s guidelines as well. T cells were then co-cultured with previously labeled WT or SLAMF7^−/−^ B cells in a 1:1 ratio with 1 × 10^6^ cells per well. Cells were cultured for 4 days, followed by staining and analysis of cells by spectral cytometry.

### ELISpot analysis

ELISPOT analysis was performed, as previously described [18, 21]. Briefly, ex vivo stimulation included the incubation of splenocytes in 100 μL of media alone (unstimulated) or media containing 6 μg/mL of MOG_35–55_ peptide. Plates were then incubated for 20 h in a 37 °C, 5% CO_2_ incubator. Development of plates was completed per the manufacturer’s protocol. Spots were counted and photographed by an automated ELISPOT reader system (Cellular Technology, Cleveland, OH). Ready-set Go IFNγ and IL-17 mouse ELISPOT kits were purchased from eBioscience (San Diego, CA).

### Anti-MOG ELISA

ELISA-based antibody assay was completed as previously described [22]. Briefly, high-binding flat-bottom 96-well plates were coated with 10 µg of MOG_35–55_ peptide per well in a volume of 100 µL and incubated overnight at 4 °C. Plates were washed with PBS–Tween (0.05%) then blocked with blocking buffer (3% bovine serum albumin) for 1 h at room temperature. Plasma was diluted in blocking buffer and added to the wells and incubated for 1 h at room temperature. Wells were then washed with PBS–Tween (0.05%) and HRP antibody (Bio-Rad) was added at 1∶2000 dilution in PBS–Tween. Tetramethylbenzidine (TMB) (Sigma-Aldrich) was added to each well and the reaction was stopped with 1N phosphoric acid. Plates were read at 450 nm in a microplate spectrophotometer.

### In vitro B cell SLAMF7 cross-linking

B cells from WT mice (*n* = 4) were isolated from spleens as described above, stained with CellTrace Violet as described above and cultured in 96-well high-binding plates at 3 × 10^5^ cells/well in Complete RPMI1640 with rmIL-2 (5 ng/mL). LPS was added to appropriate wells at 2 µg/mL. Cells were cultured for 4 days in wells coated with anti-SLAMF7 mAb (clone: 4G2) or uncoated.

### Human CSF scRNA-seq immune cell re-analysis

scRNA-seq data for six healthy controls and six MS patients were acquired from [28]. Cells with less than 200 RNA transcripts and/or more than 5800 RNA transcripts were removed, as well as cells with more than 5% of transcripts being of mitochondrial origin. Data were processed using the Seurat V4.0.4 package with the SCTransform workflow [29]. Low-quality clusters showing donor-specific biases were manually removed. SLAMF7^+^ immune cells were defined as having ≥ 1 SLAMF7 transcript (5.12% of cells were SLAMF7^+^). To predict cell–cell interactions using scRNA-seq data, Liana [30] was used to run CellphoneDB [31] on both SLAMF7^+^ and SLAMF7^−^ cells after separating out CSF immune cells from PBMCs. The CellphoneDB output from Liana was piped into the CrossTalkeR package [32] to compare cell–cell interaction networks between a network composed only of SLAMF7^+^ cells and a network composed of only SLAMF7^−^ cells. Since SLAMF7 is an adhesion and homotypic receptor, cells expressing SLAMF7 will bind to and interact with one another leading to our a priori reasoning that SLAMF7^+^ cells form a unique immune cell interaction network with possible biological implications. This approach is feasible only because SLAMF7 is a homotypic receptor.

The CCI network in Fig. 7B was constructed with the CrossTalkeR package and compares a network composed of only SLAMF7^+^ cells to one with only SLAMF7^−^ cells [32]. Cell types are nodes and are sized by the PageRank score, which is a metric measuring centrality and importance of a node to a network [32]. Edges in the CCI network are sized by the percent of predicted ligand–receptor interactions between cell types and colored based on interactions enriched in a SLAMF7^+^ network versus a SLAMF7^−^ network.

### Statistical analysis

Statistical analyses are listed in each figure legend and were carried out in GraphPad Prism V8 or the R computing environment. Cell-type frequency comparisons and differential marker expression on cell types from experiments employing spectral cytometry were statistically compared using a GLMM implemented via the diffcyt R package [33].

## Results

### SLAMF7 is protective against CNS autoimmunity and displays unique cell-type expression patterns

To assess the global impact of SLAMF7 expression and signaling on neuroinflammation, we subjected WT and SLAMF7^−/−^ mice to EAE induced with the rmMOG_1–125_ protein [34]. We found SLAMF7^−/−^ mice to be significantly more susceptible to EAE compared to WT mice (Fig. 1A, B). We also compared EAE susceptibility between WT and SLAMF7^−/−^ mice using a second, T cell-dependent EAE (rhMOG_35–55_) model and found SLAMF7^−/−^ mice to also be significantly more susceptible to EAE (Fig. 1C). Since SLAMF7 is only expressed on immune cells [14] and since immunological determinants are one of the primary factors controlling EAE susceptibility [4], we profiled the entire CNS of mice during EAE (rmMOG_1–125_) to both determine which cells express SLAMF7 and how SLAMF7 expression levels change during neuroinflammation. Employing high-dimensional single-cell spectral cytometry [16, 35, 36] we reconstructed the entire CNS immune landscape during EAE and identified 11 immune cell subsets (Fig. 1D, E) and confirmed these with exhaustive manual gating (Additional file 1: Fig. S2A). We detected SLAMF7 expression on many of the same cell types in the CNS as in the periphery including: macrophages (termed border-associated macrophages [BAMs] in the CNS), myeloid-derived cells (MdCs), pDCs, DCs, CD8^+^ T cells, NK cells, and B cells, with minimal expression on a few other subsets (Fig. 1F, G). Comparing SLAMF7 expression on various CNS immune cell subsets during steady state or active EAE neuroinflammation demonstrated that SLAMF7 expression increased on BAMs, CD8^+^ T cells, and CD4^+^ T cells during EAE (Fig. 1H), while decreasing on B cells (Fig. 1H). We also observed a significant increase in the total frequency of SLAMF7^+^ cells in the CNS during EAE compared to steady state (Fig. 1I). This is to be expected since many of the infiltrating immune cells into the CNS during EAE also express SLAMF7. Finally, while we found SLAMF7 expressed on several CNS myeloid cell types, we did not observe any SLAMF7 expression on the most populous CNS myeloid cell type, microglia (Fig. 1F, G). To confirm that microglia truly do not express SLAMF7 we imaged brains of WT mice during EAE with confocal microscopy and found punctate SLAMF7 staining on Iba1^−^ CNS immune cells but no SLAMF7 staining on any Iba1^+^ microglial cells (Additional file 1: Fig. S1A). Together, these results highlight that SLAMF7 is broadly expressed in the CNS at steady state and during neuroinflammation and plays a role in the regulation of the latter.

### SLAMF7-mediated changes in the CNS immune landscape at steady state and during EAE

**Fig. 2:**
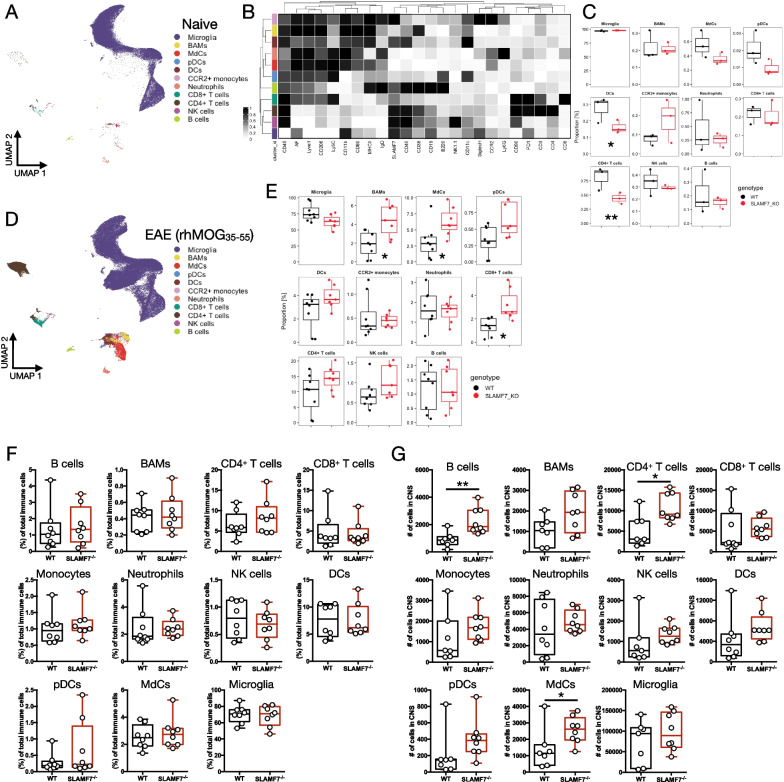
Single-cell deep CNS immune profiling of WT and SLAMF7^−/−^ mice across multiple EAE models. **A** UMAP of all CNS immune cells in the CNS of WT (*n* = 3) and SLAMF7^−/−^ (*n* = 3) mice at steady state profiled with high-dimensional single-cell spectral cytometry. **B** Heatmap of marker expression across immune cell subsets from **A**. **C** Comparison of frequencies of immune cell subsets from WT and SLAMF7^−/−^ mice at steady state. **D** UMAP of all CNS immune cells in the CNS of WT (*n* = 8) and SLAMF7^−/−^ (*n* = 7) mice during peak EAE induced with rhMOG_35–55_ profiled similarly to mice from **A**–**C**. **E** Comparison of frequencies of immune cell subsets from WT and SLAMF7^−/−^ mice in **D**. **F** Frequency of various CNS immune cell subsets between WT (*n* = 8) and SLAMF7^−/−^ mice (*n* = 8) during EAE induced with rmMOG_1–125_. **G** Total number of various immune cell subsets in the CNS of WT (*n* = 8) and SLAMF7^−/−^ mice (*n* = 8) during EAE induced with rmMOG_1–125_. Groups in **C**, **E** compared using a GLMM and representative of a single experiment. Groups in **F**, **G** compared using unpaired two-way *t*-tests and representative of two independent experiments showing similar results. *p < 0.05, **p < 0.01. BAMS, border-associated macrophages; MdCs, myeloid-derived cells

We next compared the frequency of all CNS immune cell subsets [36] between WT and SLAMF7^−/−^ mice at steady state (Fig. 2A, B) and found the frequencies of various immune cell subsets to be nearly identical, except for minor decreases in DCs and CD4^+^ T cells in SLAMF7^−/−^ mice (Fig. 2C). Extending this same analytic approach to mice subjected to EAE induced with rhMOG_35–55_, we observed significantly increased levels of CNS immune infiltration in SLAMF7^−/−^ mice, with increases specifically in BAMs, MdCs, and CD8^+^ T cells (Fig. 2D, E). No changes were observed in major splenic immune cell subsets (Additional file 1: Fig. S1G). Additionally, we also profiled CNS immune cell subsets in mice subjected to EAE induced with rmMOG_1–125_ and found no significant changes in the frequencies of various immune cell subsets (Fig. 2F), but when comparing the total number of various CNS immune cell subsets we found increased numbers of B cells, CD4^+^ T cells, and MdCs in SLAMF7^−/−^ mice (Fig. 2G).

### SLAMF7 controls CNS myeloid and B cell activation states associated with control of memory T cell responses

**Fig. 3:**
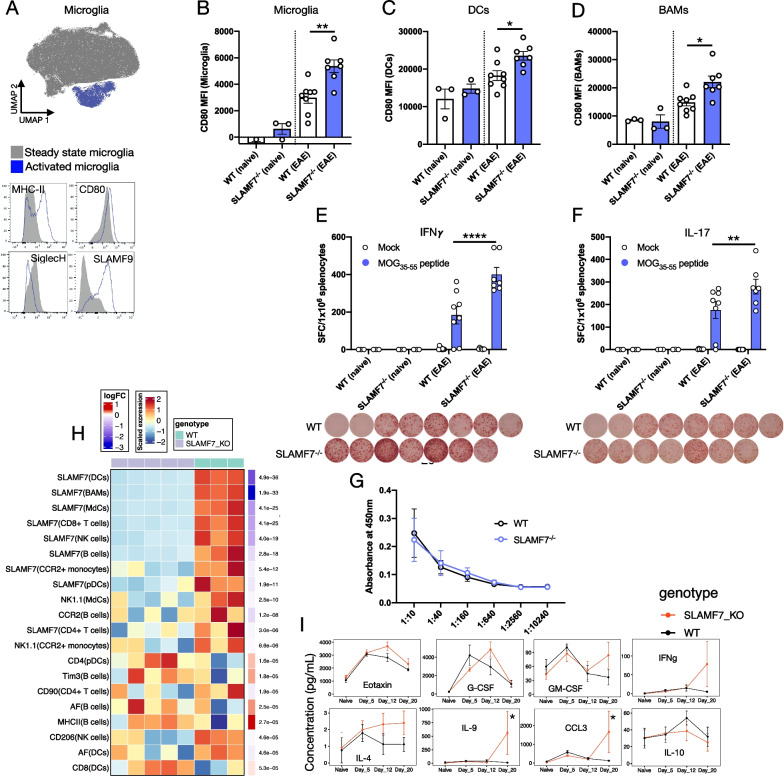
SLAMF7 restrains CNS myeloid cell activation, regulates adaptive immune responses, and controls CNS B cell activation. **A** Top, UMAP of all CNS microglia in WT mice during EAE with FlowSOM-defined clusters differentially colored. Bottom, histograms of activation marker expression on microglia subsets from above. Notably, activated microglia have very high protein-level expression of SLAMF9. **B**–**D** Expression of CD80 on microglia (**B**), DCs (**C**), and BAMs (**D**) in the CNS in WT and SLAMF7^−/−^ mice at steady state and during EAE. **E**, **F** Memory T cell ELISpot assay for IFNγ (**E**) and IL-17 (**F**) production from rhMOG_35–55_ peptide-stimulated splenocytes derived from naïve and rhMOG_35–55_-treated WT and SLAMF7^−/−^ mice. Cells were cultured without stimulation or in the presence of the rhMOG_35–55_ peptide for 24 h. Shown below each bar graph are wells containing splenocytes from EAE mice stimulated with MOG peptide. **G** ELISA of MOG_35–55_-specific total IgG using plasma derived from WT (*n* = 8) and SLAMF7^−/−^ (*n* = 7) mice subjected to EAE with rhMOG_35–55_. **H** Heatmap of differential marker expression on all CNS immune cell subsets from WT and SLAMF7^−/−^ mice during EAE (rmMOG_1–125_) with an FDR < 0.05. Adjusted *p*-values displayed on the right of each row. **I** Temporal expression of various plasma cytokines/chemokines in WT (*n* = 6) and SLAMF7^−/−^ mice (*n* = 8) during EAE induced with rmMOG_1–125_. Groups in **B**–**D** compared using a one-way ANOVA with Tukey’s multiple comparison test. Groups in **E**, **F** compared with a two-way ANOVA with FDR correction for multiple comparisons via Benjamini and Hochberg method. Groups in **H** compared with a GLMM. Groups in **I** compared using a mixed-effects model with Sidak’s post hoc test implemented through GraphPad Prism and representative of a single experiment. *p < 0.05, **p < 0.01, ****p < 0.0001. BAMS, border-associated macrophages; MdCs, myeloid-derived cells

We next profiled the activation state of various CNS-resident myeloid cell subsets to examine the role SLAMF7 plays on these cell types known to play important roles in MS and EAE [37]. We first observed two distinct microglia populations in WT EAE mice (Fig. 3A). Using high-dimensional spectral cytometry and unbiased clustering with FlowSOM, one population was identified as being highly activated as evidenced by high levels of MHC-II, CD80, and SLAMF9, along with low levels of SiglecH; all markers of microglia activation (Fig. 3A) [36]. SLAMF9 is one of the least studied members of the SLAM family, yet it has been linked to modulation of EAE susceptibility via control of pDC responses [38] and has been identified at the mRNA level as being a specific marker of disease-associated microglia (DAMs) [39]. Furthermore, we found increased expression of the myeloid cell activation marker CD80 on microglia (Fig. 3B), CNS DCs (Fig. 3C), and BAMs (Fig. 3D) in SLAMF7^−/−^ mice during EAE compared to WT mice.

Since we observed increased T cell infiltration in the CNS of SLAMF7^−/−^ mice during EAE and considering the important role of CD80 in T cell co-stimulation, we examined MOG-specific T cell responses in our model via ELISpot assay [21]. We found that SLAMF7^−/−^ mice subjected to EAE generated significantly stronger MOG peptide IFNγ (Fig. 3E) and IL-17 (Fig. 3F) memory T cell immune responses compared to WT mice. We observed no significant differences in IL-2 production in the same (data not shown). This augmented adaptive immune response did not extend to the humoral arm of the immune system as we found no significant differences in MOG peptide-specific IgG responses across genotypes (Fig. 3G).

We also compared cell surface markers on all CNS immune cell subsets between WT and SLAMF7^−/−^ mice during MOG_1–125_ EAE and found that several were significantly and differentially expressed on B cells (Fig. 3H). Specifically, SLAMF7^−/−^ B cells had higher MHC-II expression, Tim3 expression, and autofluorescence during EAE (Fig. 3H), suggesting these cells are highly activated. We also observed a number of notable instances of SLAMF7 co-expression with other regulatory receptors on CNS immune cells during EAE including: PD-1 on CD8^+^ T cells [16] (Additional file 1: Fig. S1B), CD38 on CD8^+^ T cells (Additional file 1: Fig. S1C), Tim3 on cDC1 cells (Additional file 1: Fig. S1D), and MHC-II on MdCs (Additional file 1: Fig. S1E). Importantly, CD38 expression on CNS-resident CD8^+^ T cells was decreased in SLAMF7^−/−^ mice during EAE compared to WT (Additional file 1: Fig. S1F) in line with our previous findings [16]. After observing that SLAMF7 was capable of modulating the phenotype of various CNS immune cells, we wished to determine if SLAMF7 was also capable of modulating cytokine and chemokine responses in mice during the course of EAE. We serially profiled plasma levels of multiple cytokines and chemokines in WT and SLAMF7^−/−^ mice and found a pattern of increased soluble inflammatory mediators (G-CSF, GM-CSF, IFNγ, IL-9, and CCL3) later in the disease course in SLAMF7^−/−^ mice compared to WT mice (Fig. 3I); this pattern was not conserved in mice subjected to EAE with rhMOG_35-55_ (data not shown).

### SLAMF7 does not alter the CNS-resident IL-10 immune niche during EAE

**Fig. 4:**
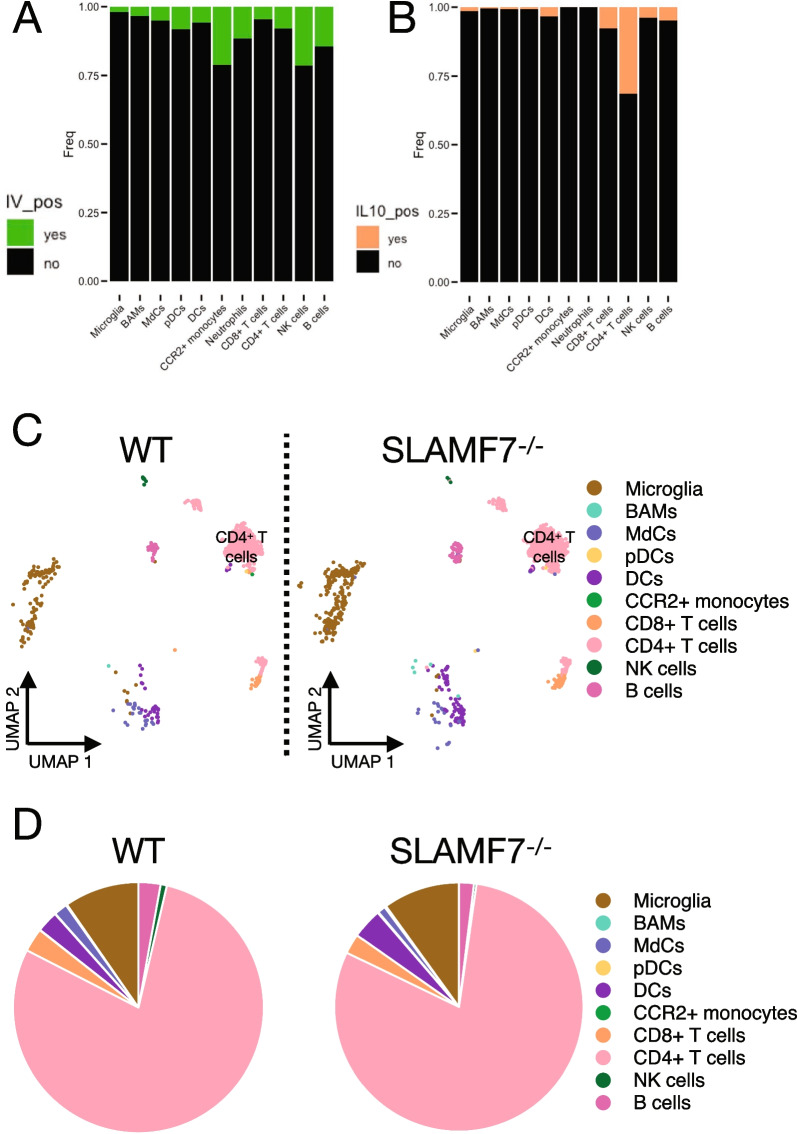
The resident CNS IL-10 immune niche in WT and SLAMF7^−/−^ mice during EAE. **A** Frequency of resident (IV^−^) versus circulating (IV^+^) CNS immune cells in WT-IL-10^GFP^ (*n* = 3) and SLAMF7^−/−^-IL-10^GFP^ (*n* = 5) mice during EAE induced with rmMOG_1–125_. **B** Frequency of IL-10 producing cells in the CNS of WT-IL-10^GFP^ (*n* = 3) and SLAMF7^−/−^-IL-10^GFP^ (*n* = 5) mice during EAE induced with rmMOG_1–125_. **C** UMAP of all CNS-resident IL-10^+^ immune cells in WT-IL-10^GFP^ and SLAMF7^−/−^-IL-10^GFP^ mice during EAE. **D** Pie charts of all CNS-resident IL-10^+^ immune cells in WT-IL-10^GFP^ and SLAMF7^−/−^-IL-10^GFP^ mice during EAE. Groups in **D** compared with a GLMM. Data in **A**–**D** representative of a single experiment. BAMS, border-associated macrophages; MdCs, myeloid-derived cells

A common mechanism of altered susceptibility to CNS autoimmunity is dysregulated immune suppressive mechanisms, such as those mediated by IL-10 [40–43]. Importantly, our previous work found that SLAMF7 signaling was capable of driving STAT3 phosphorylation [16], which is a critical driver of IL-10 production [44, 45]. Therefore, we hypothesized that lack of SLAMF7 signaling may be preventing appropriate pSTAT3-mediated IL-10 suppressive responses. To examine this, we crossed SLAMF7^−/−^ mice to IL-10^GFP^ reporter mice [46] allowing us to accurately measure IL-10 across all cell types. We induced EAE with rmMOG_1–125_ protein in WT-IL-10^GFP^ and SLAMF7^−/−^-IL-10^GFP^ mice and assessed the resident CNS immune landscape. We also performed in vivo labeling before harvest of CNS tissue to allow for identification of bona fide resident CNS immune cells [25, 36, 47]. We confirmed the majority of immune cells were indeed CNS resident, particularly for microglia and BAMs, while cell types such as CCR2^+^ monocytes and NK cells were slightly more likely to be non-resident (Fig. 4A). We therefore performed all downstream analyses using only resident CNS immune cells. Comparing the frequency of IL-10^+^ cells per immune subset, we found CD4^+^ T cells to contain the largest fraction of IL-10^+^ cells (Fig. 4B). We next reconstructed the CNS-resident IL-10 immune landscape (Fig. 4C) and found CD4^+^ T cells and microglia to be the predominant sources of IL-10 during EAE [25], and that this was consistent between IL-10^GFP^ and SLAMF7^−/−^-IL-10^GFP^ mice (Fig. 4D). These results suggest that SLAMF7 does not alter the CNS IL-10 immune niche during EAE and argues in favor of alternative mechanisms explaining enhanced SLAMF7^−/−^ mouse susceptibility to EAE.

### Deep phenotyping of B cells at steady state and during EAE reveals plasma cell and T2 B cell alterations in SLAMF7^−/−^ mice

**Fig. 5:**
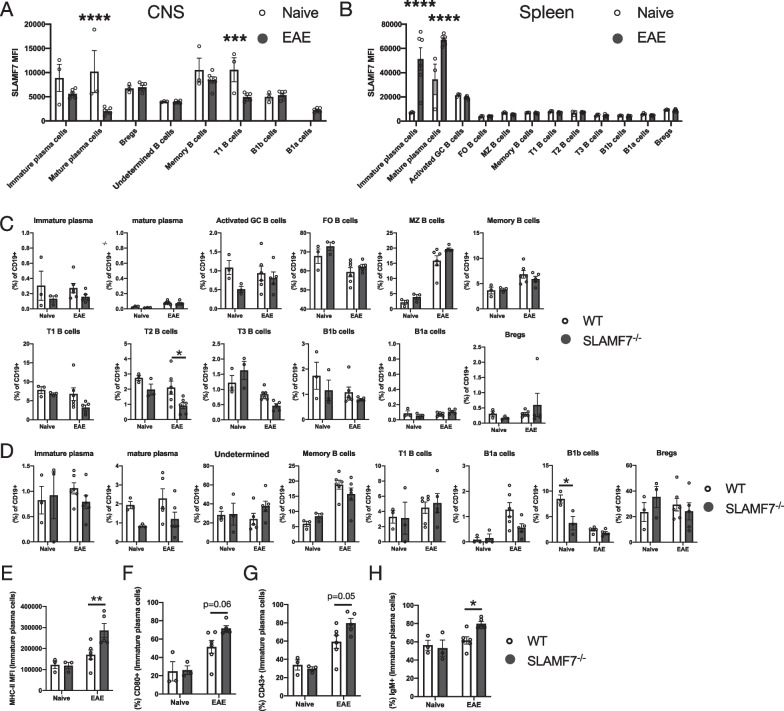
Deep phenotyping of splenic and CNS B cell subsets in WT and SLAMF7^−/−^ mice. **A**, **B** SLAMF7 expression level across CNS (**A**) and splenic (**B**) B cell subsets at steady state and during active EAE neuroinflammation in WT mice. **C** Frequency of various B cell subsets from total splenic B cells in WT and SLAMF7^−/−^ mice at steady state and during EAE. **D** Frequency of various B cell subsets from total CNS B cells in WT and SLAMF7^−/−^ mice at steady state and during EAE. **E** MHC-II expression on splenic immature plasma cells. **F** CD80 expression on splenic immature plasma cells. **G** CD43 expression on splenic immature plasma cells. **H** IgM expression on splenic immature plasma cells. Data in **A**–**H** representative of a single experiment. Groups in **A**, **B** compared with multiple unpaired two-way *t*-tests with a Holm–Sidak correction for multiple comparisons. Groups in **C**–**H** compared with a two-way ANOVA with Sidak’s test for multiple comparisons. FO, follicular; MZ, marginal zone; Bregs, regulatory B cells

We examined how SLAMF7 signaling modulated B cell responses in our model due to the fact that we saw a more robust difference in EAE clinical scores between WT and SLAMF7^−/−^ mice when using a B cell-dependent model of EAE (rmMOG_1–125_ protein [48–51]), observed increased numbers of B cells in the CNS of SLAMF7^−/−^ mice in this model (Fig. 2G), and because B cells were the only CNS immune cell subset where SLAMF7 expression was decreased during EAE compared to steady state (Fig. 3H). To comprehensively profile B cells, we utilized our previously described high-dimensional spectral cytometry B cell profiling approach (Additional file 1: Fig. S3A and Additional file 2: Table S1) [25] to analyze both splenic and CNS B cells from WT and SLAMF7^−/−^ mice at steady state and during EAE (rmMOG_1–125_). We found changes in SLAMF7 expression across various B and plasma cell subsets in the CNS, observing decreased SLAMF7 expression on mature plasma cells and T1 B cells during EAE (Fig. 5A). However, in the spleens of the same mice we noted increased SLAMF7 expression on immature and mature plasma cells during EAE (Fig. 5B). Comparing the frequency of the 12 splenic B cell subsets between WT and SLAMF7^−/−^ mice, we observed a minor decrease in T2 B cells in SLAMF7^−/−^ mice during EAE compared to WT mice (Fig. 5C). Similarly, in the CNS we only observed a small decrease in the frequency of B1b (innate-like) cells in SLAMF7^−/−^ mice at steady state compared to WT mice (Fig. 5D). Together, these data suggest that SLAMF7 expression does not globally remodel either the CNS or splenic B cell compartments at steady state or during active neuroinflammation.

Additionally, we found increased expression of the activation markers MHC-II (Fig. 5E), CD80 (Fig. 4F), and CD43 (Fig. 4G) on splenic immature plasma cells of SLAMF7^−/−^ mice only during EAE compared to WT. We also observed increased expression of IgM on SLAMF7^−/−^ splenic immature plasma cells only during EAE (Fig. 5H) which is intriguing since IgM^+^ plasma cells are known to retain a functional BCR and are potent cytokine producers [52]. We also noted a number of phenotypic differences on T2 and MZ B cells including: decreased CD1d expression on MZ (Additional file 1: Fig. S3B) and T2 (Additional file 1: Fig. S3C) B cells from SLAMF7^−/−^ mice and increased MHC-II (Additional file 1: Fig. S3D) and GL7 (Additional file 1: Fig. S3E) on T2 B cells from SLAMF7^−/−^ mice during EAE. Since T2 B cells are recognized as a precursor to MZ B cells [53, 54], the latter of which expand greatly during EAE (Fig. 5C), our findings of increased activation in these cells suggest SLAMF7 may modulate responses of this cell lineage early during their development. Together, these results suggest that SLAMF7 expression does not globally remodel either the CNS or splenic B cell compartments at steady state or during active neuroinflammation, but rather suggests that SLAMF7 is linked to activation and modulation of B cell states.

### SLAMF7 intrinsically regulates B cell activation and B cell-induced T cell responses to control EAE severity

**Fig. 6:**
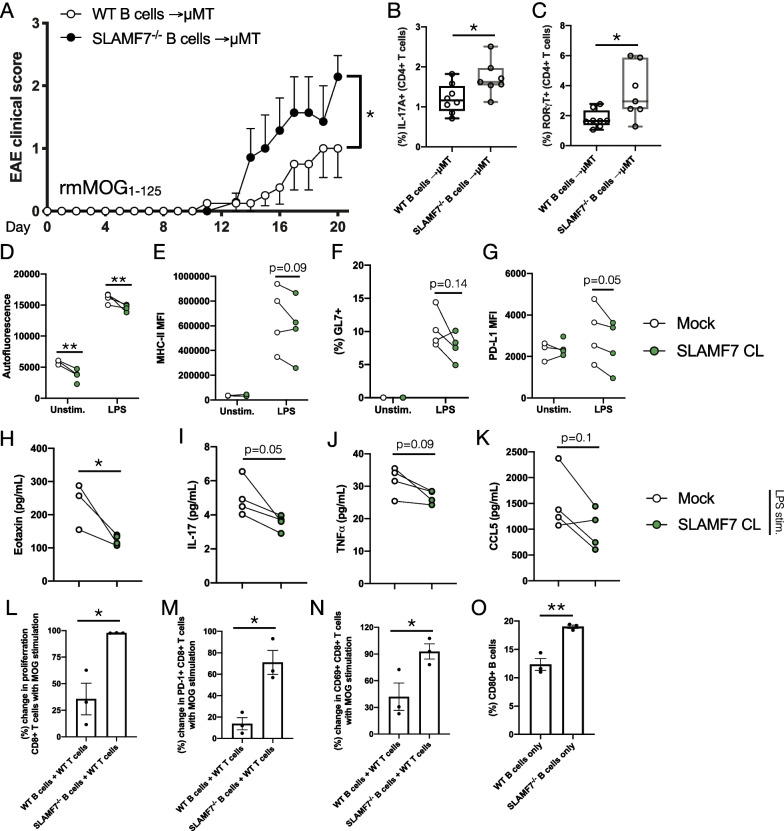
SLAMF7 tempers B cell activation to restrict T cell activation resulting in concomitant modulation of EAE severity. **A** Clinical scores of μMT mice reconstituted with either WT B cells (*n* = 8) or SLAMF7^−/−^ B cells (*n* = 7) and subjected to EAE induced with rmMOG_1–125_. **B** Expression of IL-17A in peripheral blood CD4^+^ T cells from mice in **A**. **C** Expression of RORγt in peripheral blood CD4^+^ T cells from mice in **A**. **D**–**K** Isolated splenic B cells from WT mice (*n* = 4) were cultured for 4 days with SLAMF7 activation (SLAMF7 cross-linking) or without (mock) and surface and soluble markers of B cell activation were assessed. Changes in autofluorescence (**D**), MHC-II MFI (**E**), GL7 expression (**F**), and PD-L1 MFI (**G**) on B cells cultured with or without SLAMF7 activation. Changes in the levels of cytokines and chemokines from supernatant of B cells cultured with and without SLAMF7 activation including Eotaxin (**H**), IL-17 (**I**), TNF⍺ (**J**), and CCL5 (**K**). **L**–**O** EAE was induced in WT mice and splenic CD3^+^ T cells were harvested on day 60. T cells were labeled with CellTrace Violet and co-cultured with splenic B cells isolated from either WT or SLAMF7^−/−^ mice for 4 days in the presence and absence of MOG_35–55_ peptide. **L** Percent change in the frequency of proliferating CD8^+^ T cells comparing unstimulated cells to MOG_1–125_ stimulated cells. **M** Percent change in PD-1 expression on CD8^+^ T cells comparing unstimulated cells to MOG_35–55_ stimulated cells. **N** Percent change in CD69 expression on CD8^+^ T cells comparing unstimulated cells to MOG_35–55_ stimulated cells. **O** Frequency of CD80^+^ unstimulated B cells isolated from the spleens of WT or SLAMF7^−/−^ mice. Data in **A**–**O** representative of an individual experiment. Groups in **A** compared with two-way ANOVA with Sidak’s correction for multiple comparisons. Groups in **B**, **C** and **H**–**O** compared with a two-way unpaired *t*-test. Data in **D**–**G** compared with a mixed-effects model with FDR correction for multiple comparisons via Benjamini and Hochberg method. *p < 0.05, **p < 0.01

We next wished to confirm that SLAMF7 expression and signaling on B cells could modulate EAE severity in vivo. To accomplish this, we adoptively transferred B cells isolated from WT or SLAMF7^−/−^ mice into μMT mice (genetically deficient in B cells [55]) and induced EAE with rmMOG_1–125_ protein as previously described [25]. We found that μMT mice reconstituted with SLAMF7^−/−^ B cells experienced significantly increased incidence of EAE symptoms compared to mice reconstituted with WT B cells (Fig. 6A), confirming an intrinsic role for SLAMF7 expression in B cells as responsible for regulating CNS autoimmunity. We assessed the phenotype of T cells in the peripheral blood of these mice and found a higher frequency of IL-17A^+^ CD4^+^ T cells and RORγt^+^ CD4^+^ T cells (Fig. 6B, C and Additional file 1: Fig. S4A) in mice reconstituted with SLAMF7^−/−^ B cells, suggesting SLAMF7 expression on B cells might regulate the development of Th17 cells. We further confirmed no difference in the CNS CD4/CD8 T cell ratio (Additional file 1: Fig. S4B) or frequency of CNS B cells in these mice (Additional file 1: Fig. S4C).

To evaluate how SLAMF7 intrinsically regulates B cell responses we cultured WT splenic B cells in vitro in the presence or absence of SLAMF7 receptor activation (via receptor cross-linking [16]) and evaluated cell surface B cell activation markers via spectral cytometry. We found decreased autofluorescence (a proxy for lymphocyte activation) (Fig. 6D), MHC-II (Fig. 6E), GL7 (Fig. 6F), and PD-L1 expression (Fig. 6G) on SLAMF7-activated B cells compared to unstimulated B cells. Examination of soluble factors in supernatant of cultures of SLAMF7-stimulated B cells revealed decreased levels of Eotaxin (Fig. 6H), IL-17 (Fig. 6I), TNF⍺ (Fig. 6J), and CCL5 (Fig. 6K) in B cells with SLAMF7 activation compared to unstimulated cells, revealing an inhibitory role for SLAMF7 in B cells. While changes in some of these surface and soluble activation markers were minimal, together they suggest SLAMF7 signaling is capable of tempering B cell activation in a mild manner.

To determine what role SLAMF7 expression on B cells plays in regulating T cell responses, we set up an in vitro co-culture model consisting of T cells isolated from WT mice at peak EAE severity, combined with either WT or SLAMF7^−/−^ B cells isolated from naïve mice, along with stimulation using MOG peptide. Using this model, we were able to assess the contribution of SLAMF7 expression on B cells to antigen-induced T cell proliferation and found that SLAMF7 expression on B cells restrains antigen-induced CD8^+^ T cell proliferation (Fig. 6L and Additional file 1: Fig. S4D). Similarly, SLAMF7 expression on B cells also decreased antigen-induced PD-1 expression on CD8^+^ T cells (Fig. 6M) and CD69 expression on CD8^+^ T cells (Fig. 6N) (no significant changes found in CD4^+^ T cell phenotypes (data not shown)). Fittingly, we also found that SLAMF7^−/−^ B cells express more CD80 compared to WT B cells (Fig. 6O). Together, these results suggest that in the absence of SLAMF7, B cells are more prone to activation and preferentially induce CD8^+^ T cell activation, possibly via MHC-II, PD-L1, CD80, and/or direct SLAMF7 ligation mechanisms.

### B cells orchestrate cell–cell interactions between SLAMF7-expressing cells in human CSF

While our above studies identified a functional role for SLAMF7 in the regulation of CNS autoimmunity in mice, the importance of SLAMF7 in the human CNS immune landscape, particularly as it relates to MS, remains undefined. Since SLAMF7 is a self-ligand, this implies that all immune cells in a given niche expressing SLAMF7 may form a unique interactome. We leveraged this unique feature of SLAMF7 biology to reconstruct a SLAMF7-specific cell–cell interaction (CCI) network of human CSF immune cells from healthy controls and MS patients.

**Fig. 7:**
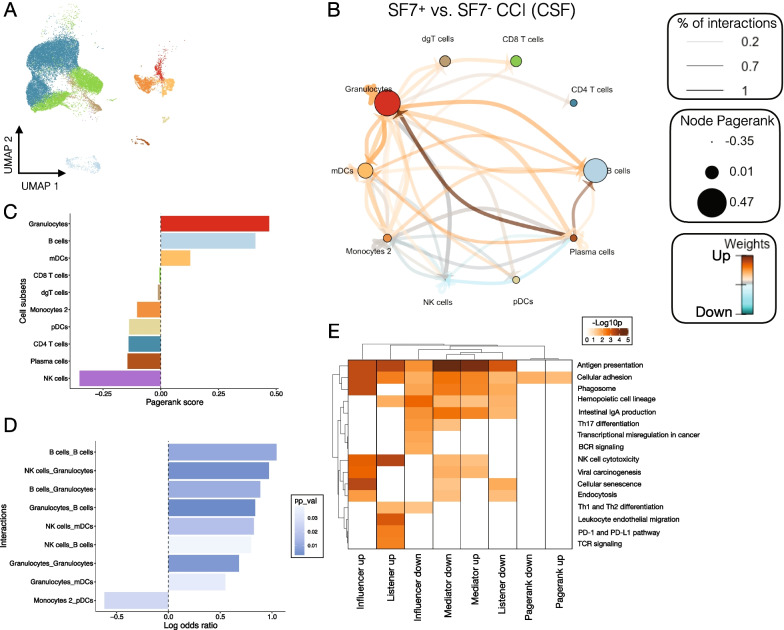
B cells function as central hub orchestrating cell–cell interactions between SLAMF7^+^ cells in the CNS. **A** UMAP of 31,063 immune cells from the CSF of six healthy controls and six MS patients reanalyzed from [28]. **B** Cell–cell interaction (CCI) network comparing differential cellular interaction networks between SLAMF7^+^ and SLAMF7^−^ immune cells from **A**. Each node is a cell type which is scaled according to its PageRank score (centrality in SLAMF7^+^ vs. SLAMF7^−^ networks). Arrows connecting nodes show directionality of cell–cell interactions, are sized based on the percent of interactions comparing SLAMF7^+^ to SLAMF7^−^ networks, and are colored based on whether the interactions are up- or down-regulated in SLAMF7^+^ vs. SLAMF7^−^ networks. **C** Bar plot of PageRank scores from the CCI network in **B** highlighting B cells and granulocytes as being the most important cell types in SLAMF7^+^ immune cell interaction networks compared to SLAMF7^−^ networks. **D** Bar plot showing the log odds ratio for all significant cell–cell interactions between SLAMF7^+^ vs. SLAMF7^−^ networks. A positive log odds ratio indicates that interaction is enriched in a network composed of SLAMF7^+^ immune cells compared to SLAMF7^−^ immune cells. **E** Heatmap of differentially regulated KEGG pathways for the topological measures used to calculate the CCI network in **B**. Influencers are the outgoing signals from each cell type, listeners are the incoming signals to each cell type, moderators measure the importance of a cell type to mediate communication between cell types, and PageRank is the importance of each cell type to orchestrating cell–cell communications in a network [32]. dgT cells, δγ T cells

Previously published scRNA-seq data [28] of 31,000 CSF immune cells from healthy controls and MS patients were re-analyzed (see “Materials and methods”) to reconstruct the CSF immune landscape and 11 distinct immune cell subsets were identified (Fig. 7A). *SLAMF7* expression on all immune cells was analyzed and cells were identified as being *SLAMF7*^+^ or *SLAMF7*^*−*^. *SLAMF7*^+^ and *SLAMF7*^*−*^ cells were separately run through the CellPhoneDB program in order to perform cell–cell interaction (CCI) prediction based on ligand–receptor pair expression from the scRNA-seq data [31]. Next, the *SLAMF7*^+^ network was compared to the *SLAMF7*^*−*^ network using CrossTalkeR [32] and a comparative CCI network was constructed (Fig. 7B). Each node in this network is an immune cell subset and is sized according to its relative importance in orchestrating cell–cell interactions in a *SLAMF7*^+^ vs. *SLAMF7*^*−*^ immune cell network (via PageRank scores) (Fig. 7B, C). From this we can appreciate that B cells and granulocytes are coordinating significantly more cell–cell interactions between other *SLAMF7*^+^ immune cells in a *SLAMF7*^+^ vs. *SLAMF7*^*−*^ CSF immune cell network. The arrows (edges) connecting nodes in Fig. 7B represent the frequency of ligand–receptor interactions between cell types and are colored red if those interactions are upregulated in a network of *SLAMF7*^+^ vs. *SLAMF7*^*−*^ immune cells and blue if the reverse is true. From this, and the corresponding log odds ratios of cell–cell interaction probabilities (Fig. 7D), we can appreciate how in the *SLAMF7*^+^ CSF immune niche B cells exhibit promiscuous interactions with multiple other immune cell subsets such as: other B cells, granulocytes, and NK cells (Fig. 7D). Outgoing signaling from plasma cells to both B cells and granulocytes is enriched in the *SLAMF7*^+^ network presumably because all plasma cells express SLAMF7 [14]. Finally, we assessed each of the topological measures [32] across our CCI network for enrichment of various KEGG pathways to help better highlight the biological significance of SLAMF7 signaling in the CSF. We found antigen processing and presentation, cell adhesion, the phagosome, and endocytosis to be increased in cells sending out signals (influencers) in the *SLAMF7*^+^ network (Fig. 7E). In agreement with our previous data showing a link between SLAMF7 expression on B cells with Th17 differentiation (Figs. 3F, 6B, C), we observed enrichment in the Th17 cell differentiation KEGG pathway in mediator type cells (topological network measure of how much a cell type talks to other cells) and influencer cells in *SLAMF7*^*−*^ networks (Fig. 7E). Together, these results highlight the important role B cells play in coordinating cell–cell cross talk among *SLAMF7*^+^ cells in the CNS.

## Discussion

The increased susceptibility to EAE we observe in SLAMF7^−/−^ mice links well to GWAS studies showing an association of polymorphisms in *SLAMF7* with MS [4, 11, 12]. It supports a role for SLAMF7 in dampening over-zealous, pathology-inciting immune responses, which when perturbed, lead to MS susceptibility. This fits with our current understanding of SLAMF7, since most immune cells that express SLAMF7 do not express the EAT-2 adaptor, or downregulate its expression upon SLAMF7 activation, meaning that SLAMF7 signaling on most immune cells is expected to be inhibitory or anti-inflammatory [14, 15].

Using both the B cell and T cell-dependent modes of EAE, we observed increased susceptibility of SLAMF7^−/−^ of EAE compared to WT mice. Differences across our models may be related to the differing pathological mechanisms underlying each model [56] and, considering each of the immune cell types significantly increased in SLAMF7^−/−^ mice in the rhMOG_35–55_ model had high SLAMF7 expression (Fig. 1G), may indicate a direct role for SLAMF7 in CNS trafficking and residency. Conversely, results from our rmMOG_1–125_ EAE model where we observed increased numbers of immune cell subsets with low SLAMF7 expression (Fig. 1G), point toward a more indirect role for SLAMF7 in regulating neuroinflammation, possibly via effects occurring in the periphery during early auto-antigen presentation.

After profiling the expression of SLAMF7 across the entire CNS immune landscape, we found SLAMF7 expression to closely mirror that of immune cells in the periphery [13, 14], with the notable exception of B cells where a decrease in SLAMF7 expression was observed during EAE. This decrease in SLAMF7 expression in the setting of a highly inflammatory environment is contrary to prior studies exploring SLAMF7, as this receptor is quite responsive to inflammatory stimuli and typically increases in expression in these settings [15, 16, 57]. This points to a potential role for SLAMF7 signaling in modulating B cell responses in the setting of CNS inflammation also evidenced from B cell changes detected in our unbiased neuroimmune phenotyping panel. Since our previous studies have shown that activating SLAMF7 can lead to its down-regulation [15, 23], this may indicate that SLAMF7 ligation and downstream signaling are occurring in B cells during EAE neuroinflammation. In support of this, and relevant to our studies here showing a role for SLAMF7 expression on B cells in the setting of neuro-autoimmunity, a recent study has shown that SLAMF7 signaling on B cells is inhibitory and antagonizes BCR activation [58].

The association between SLAMF7 expression/signaling in B cells and susceptibility to CNS autoimmunity is supported by our adoptive transfer studies and is particularly exciting considering how the new B cell-depleting therapies are changing the landscape of contemporary MS treatment [8, 59]. How B cells contribute to MS pathogenesis is one of the most pressing questions in the MS field, and a number of theories have been proposed and studied, yet none has emerged as a unifying mechanism [59, 60]. Interestingly, there is also an FDA-approved therapy targeting SLAMF7, elotuzumab [61]. Elotuzumab is approved for the treatment of multiple myeloma and functions primarily via antibody-dependent cellular cytotoxicity, but has also been shown to have SLAMF7 agonistic activity [62]. Considering our results in a pre-clinical model of MS, it would be interesting to see how a SLAMF7 agonist would perform in MS patients, especially in combination with other immuno-modulatory agents.

An interesting discovery from our in-depth investigations of B cells was the ability of SLAMF7 to temper B cell activation, as evidenced from both increased activation makers in SLAMF7^−/−^ B cells and vice versa in B cells subjected to SLAMF7 activation. This effect was consistent across both of our complementary animal models, specifically in regard to autofluorescence and MHC-II expression. Our finding that SLAMF7 signaling on B cells down-regulates their production of Eotaxin (CCL11) is interesting considering plasma Eotaxin levels are known to be upregulated in individuals with a more severe form of MS [63]. Consequently, future studies assessing the linkage of the rs983494 *SLAMF7* SNP with primary progressive and other MS subsets will be particularly interesting.

The ability of SLAMF7 to regulate the numbers and activation state of the T2 B cell subset make this one potential mechanism explaining the genetic association between SLAMF7 and MS. T2 B cells are a subset of transitional B cells which are bone marrow-derived and serve as precursors to mature B cells [64]. Lower numbers of these cells in the periphery as compared to other B cell subsets makes study of these cells difficult, but mounting evidence suggests that they are more than merely a transitional stage in B cell development, and that they may play an important role in the immunological responses to infectious agents and autoimmunity [64]. The increased activation level of T2 B cells (as evidenced by MHC-II and GL7 expression) from SLAMF7^−/−^ mice during EAE, paired with the decreased frequency of these cells, may indicate these cells are undergoing activation-induced cell death due to the absence of SLAMF7. Furthermore, MS patients tend to have lower levels of transitional B cells along with elevated CD80 expression on those cells [65]; very similar to what we observe in SLAMF7^−/−^ mice. With a relative decrease of 50% compared to WT mice, this finding of decreased transitional B cells existing in a primed/activated state may help explain why μMT mice reconstituted with SLAMF7^−/−^ B cells have an exaggerated Th17 response; a phenomenon known to be pathogenic in MS/EAE [66]. This is because previous studies have shown that T2 B cells can suppress Th17 development and IL-17 production [64]. Furthermore, transitional B cells have also been found to be capable of inhibiting CD8^+^ T cell responses which similarly may explain results from our B/T cell co-culture studies showing increased CD8^+^ T cell activation in conditions containing SLAMF7^−/−^ B cells (which presumably have fewer transitional B cells) [64]. However, a specific role for transitional B cells in MS/EAE has yet to be defined [64], thus further studies of the role of SLAMF7 on B cells are needed to address this.

Another mechanism potentially contributing to increased EAE incidence in SLAMF7^−/−^ mice is the decreased CD1d expression on MZ and T2 B cells. CD1d is a cell surface receptor which recognizes lipid antigens and can present them to invariant NKT (iNKT) cells to induce activation of these cells [67]. The decreased CD1d expression on B cell subsets in SLAMF7^−/−^ mice suggests that iNKT cell activation will be impaired during EAE in these mice [68, 69]. Since iNKT cell activation has been generally shown to be protective in EAE [67, 70], this mechanism may help explain both the increased EAE incidence in SLAMF7^−/−^ mice and the increased incidence in μMT mice reconstituted with SLAMF7^−/−^ B cells.

How the expression of SLAMF7 and other SLAM family receptors affect cell–cell communication in the CNS is currently unknown and represents an attractive area of research considering the homotypic nature of these receptors and their ability to broadly modify immune cell functions [13, 14]. We have begun to address this through a computational approach for predicting cell–cell interactions via ligand and receptor expression from scRNA-seq data of human CSF immune cells [28]. Unexpectedly, our results aligned with our EAE studies, in that our analysis of human CSF immune cells showed B cells to be one of the most important cell types for coordinating cell–cell interactions among SLAMF7^+^ CSF immune cells. How precisely SLAMF7^+^ B cells interact with other SLAMF7^+^ immune cells in the CNS to modulate immunity remains to be seen, but may involve cell adhesion and/or regulation of Th17 cell differentiation based on our murine and computational studies.

Our finding that microglia do not express SLAMF7 was unexpected considering most other myeloid subsets express SLAMF7. There are a number of possible explanations for this, one of which concerns the origin of microglia. Unlike other myeloid cells, microglia have a unique ontogeny, arising from the yolk sac during development [71, 72], which may play a role in the unique cellular programs present in microglia compared to other myeloid cells. Whether this effect is conserved for other SLAM receptors and the potential functional consequences of this are interesting questions for future investigations. Additionally, recent work has shown that a subset of CNS-resident border-associated macrophages (BAMs) are yolk sac-derived [73, 74], yet BAMs express SLAMF7. If different subsets of BAMs, from disparate ontogenies, differentially express SLAMF7 and other SLAM receptors, and what programs are responsible for this, will be important questions to answer as we begin to define the roles of SLAM receptors across the CNS immune landscape. Finally, by validating that SLAMF9 is expressed on the cell surface of activated microglia at the protein level, this opens up the opportunity to both use SLAMF9 as a marker of DAMs and as a potential therapeutic target [75].

## Conclusions

Here we have shown, using multiple murine models of MS, that the immune cell receptor SLAMF7 regulates susceptibility to CNS autoimmunity through B cells and memory T cell responses. This provides a mechanistic explanation for the genetic association between a SNP in the SLAMF7 promoter and susceptibility to MS, and provides a foundation on which future studies aimed at modulating SLAM family receptors to treat autoimmune diseases may build from.

## Supplementary Information

**Additional file 1: Figure S1.** (A) Confocal imaging of SLAMF7 and Iba1 on mouse brain tissue during peak EAE. Magnifications 1 and 2 highlight regions with SLAMF7^+^ Iba1^−^ non-microglial cells and SLAMF7^−^ Iba1^+^ microglia, respectively. (B–E) Notable co-expression patterns of SLAMF7 with other regulatory markers on various CNS immune cell subsets during EAE. (B) Co-expression of SLAMF7 and PD-1 on CD8^+^ T cells, (C) SLAMF7 co-expression with CD38 on CD8^+^ T cells, (D) co-expression of SLAMF7 and Tim-3 on cDC1 cells, (E) co-expression of MHC-II and SLAMF7 on MdCs. (F) CD38 expression on CNS CD8 + T cells. (G) Splenic immune cell subset frequencies in naive WT (*N * = 3), naive SLAMF7^−/−^ (*n* = 3), WT EAE (*n* = 8), and SLAMF7^−/−^ EAE (*n* = 7) mice. Groups in (F, G) compared with a two-way ANOVA with FDR correction for multiple comparisons via Benjamini and Hochberg method. *p < 0.05, **p < 0.01. MdCs, myeloid-derived cells. **Figure S2.** Gating schemes. (A) Gating scheme used to manually annotate nearly all CNS immune cell subsets (used in (Figs. 1H and 2F, G)). This gating scheme is able to identify approximately 98% of all CD45^+^ CNS immune cells. Put. Microglia, putative microglia; BAM, border-associated macrophage; MdCs, myeloid-derived cells. **Figure S3.** Gating and additional analyses of B cell deep phenotyping (related to Fig. 5). (A) Gating scheme used to clean up datasets and manually annotate all CNS and splenic B cell subsets from high dimensional B cell profiling experiments. (B) CD1d expression on MZ B cells. (C) CD1d expression on T2 B cells. (D) MHC-II expression on T2 B cells. (E) GL7 expression on T2 B cells. Groups in (B–E) compared with a two-way ANOVA with Sidak’s test for multiple comparisons. FO, follicular; MZ, marginal zone; Bregs, regulatory B cells. *p < 0.05, **p  < 0.01, ***p < 0.001. **Figure S4.** Gating schemes (related to Fig. 6). (A) Gating scheme used to identify IL-17A^+^ and RORgt^+^ CD4^+^ T cells in µMT mice (used in (Fig. 6B, C)). (B) CD4/CD8 T cell ratio in the CNS of mice µMT mice. (C) Frequency of B cells in the CNS of µMT mice. (D) Gating scheme used to measure CD8^+^ T cell phenotypes from co-culture experiments (used in (Fig. 6L–N)).

**Additional file 2: Table S1.** Antibody panels used.

## Data Availability

All raw data are available upon reasonable request. Analysis code can be found at: https://github.com/poconnel3/OConnell_et_al_SLAMF7_EAE_paper_code.
