# Supplementary material for: SLAMF7 modulates B cells and adaptive immunity to regulate susceptibility to CNS autoimmunity
Source: J Neuroinflammation. 2022 Oct 3;19:241. doi: 10.1186/s12974-022-02594-9 (PMC9533612; doi:10.1186/s12974-022-02594-9)
Supplement: Supplementary file 1 — Additional file 1: Figure S1. (A) Confocal imaging of SLAMF7 and Iba1 on mouse brain tissue during peak EAE. Magnifications 1 and 2 highlight regions with SLAMF7+ Iba1− non-microglial cells and SLAMF7− Iba1+ microglia, respectively. (B–E) Notable co-expression patterns of SLAMF7 with other regulatory markers on various CNS immune cell subsets during EAE. (B) Co-expression of SLAMF7 and PD-1 on CD8+ T cells, (C) SLAMF7 co-expression with CD38 on CD8+ T cells, (D) co-expression of SLAMF7 and Tim-3 on cDC1 cells, (E) co-expression of MHC-II and SLAMF7 on MdCs. (F) CD38 expression on CNS CD8 + T cells. (G) Splenic immune cell subset frequencies in naive WT (N = 3), naive SLAMF7−/− (n = 3), WT EAE (n = 8), and SLAMF7−/− EAE (n = 7) mice. Groups in (F, G) compared with a two-way ANOVA with FDR correction for multiple comparisons via Benjamini and Hochberg method. *p < 0.05, **p < 0.01. MdCs, myeloid-derived cells. Figure S2. Gating schemes. (A) Gating scheme used to manually annotate nearly all CNS immune cell subsets (used in (Figs. 1H and 2F, G)). This gating scheme is able to identify approximately 98% of all CD45+ CNS immune cells. Put. Microglia, putative microglia; BAM, border-associated macrophage; MdCs, myeloid-derived cells. Figure S3. Gating and additional analyses of B cell deep phenotyping (related to Fig. 5). (A) Gating scheme used to clean up datasets and manually annotate all CNS and splenic B cell subsets from high dimensional B cell profiling experiments. (B) CD1d expression on MZ B cells. (C) CD1d expression on T2 B cells. (D) MHC-II expression on T2 B cells. (E) GL7 expression on T2 B cells. Groups in (B–E) compared with a two-way ANOVA with Sidak’s test for multiple comparisons. FO, follicular; MZ, marginal zone; Bregs, regulatory B cells. *p < 0.05, **p < 0.01, ***p < 0.001. Figure S4. Gating schemes (related to Fig. 6). (A) Gating scheme used to identify IL-17A+ and RORgt+ CD4+ T cells in µMT mice (used in (Fig. 6B, C)). (B) CD4/CD8 T cell ratio in t [file 12974_2022_2594_MOESM1_ESM.pdf]

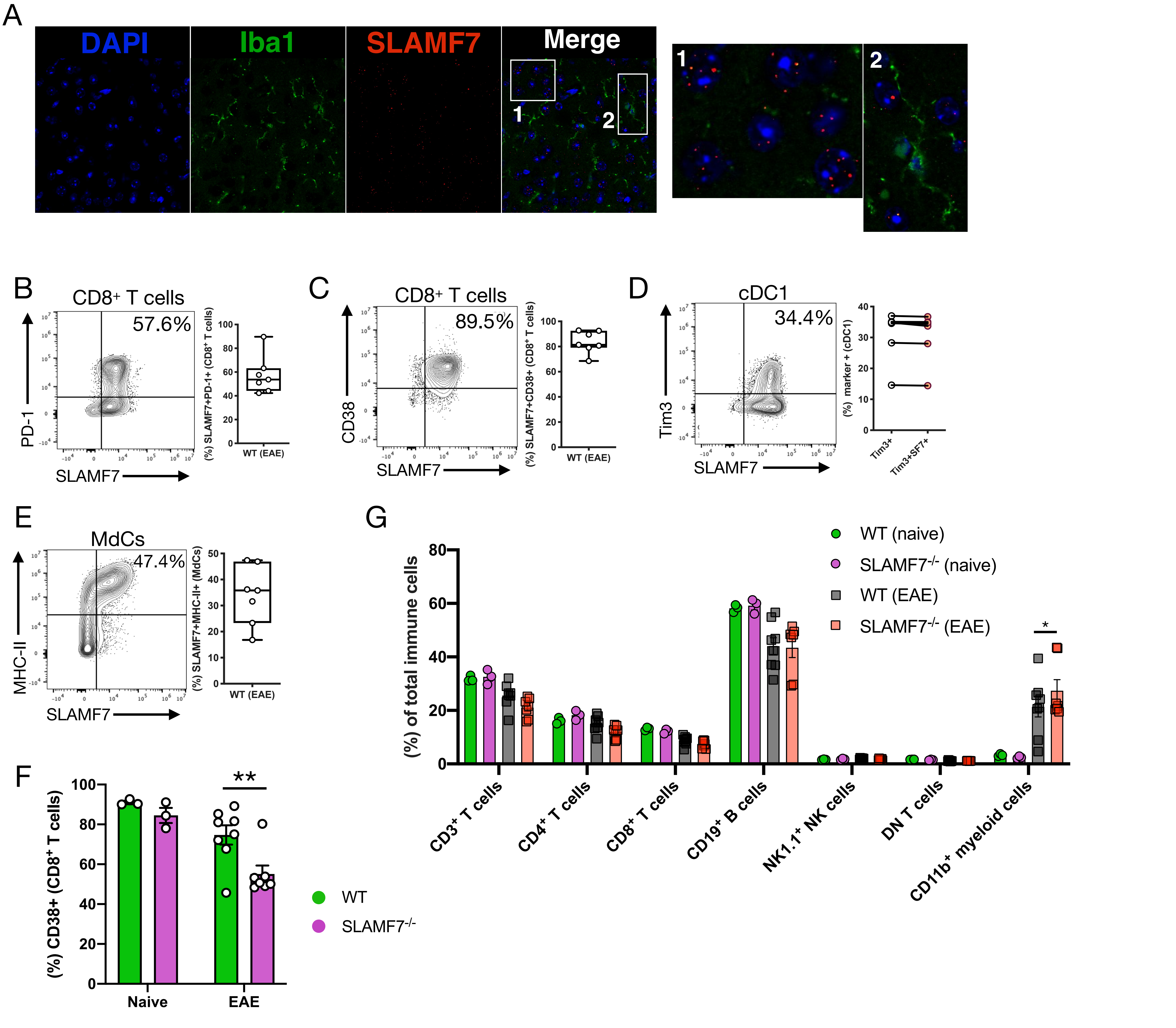

Figure S1. (A) Confocal imaging of SLAMF7 and Iba1 on mouse brain tissue during peak EAE. Magnifications 1 and 2 highlight regions with SLAMF7+ Iba1- non-microglial cells and SLAMF7- Iba1+ microglia, respectively. (B-E) Notable co-expression patterns of SLAMF7 with other regulatory markers on various CNS immune cell subsets during EAE. (B) Co-expression of SLAMF7 and PD-1 on CD8+ T cells, (C) SLAMF7 co-expression with CD38 on CD8+ T cells, (D) co-expression of SLAMF7 and Tim-3 on cDC1 cells, (E) co-expression of MHC-II and SLAMF7 on MdCs. (F) CD38 expression on CNS CD8+ T cells. (G) Splenic immune cell subset frequencies in naive WT (N=3), naive SLAMF7-/- (n=3), WT EAE (n=8), and SLAMF7-/- EAE (n=7) mice. Groups in (F, G) compared with a two-way ANOVA with FDR correction for multiple comparisons via Benjamini and Hochberg method. *$p<0.05$, **$p<0.01$. MdCs, myeloid-derived cells.

A

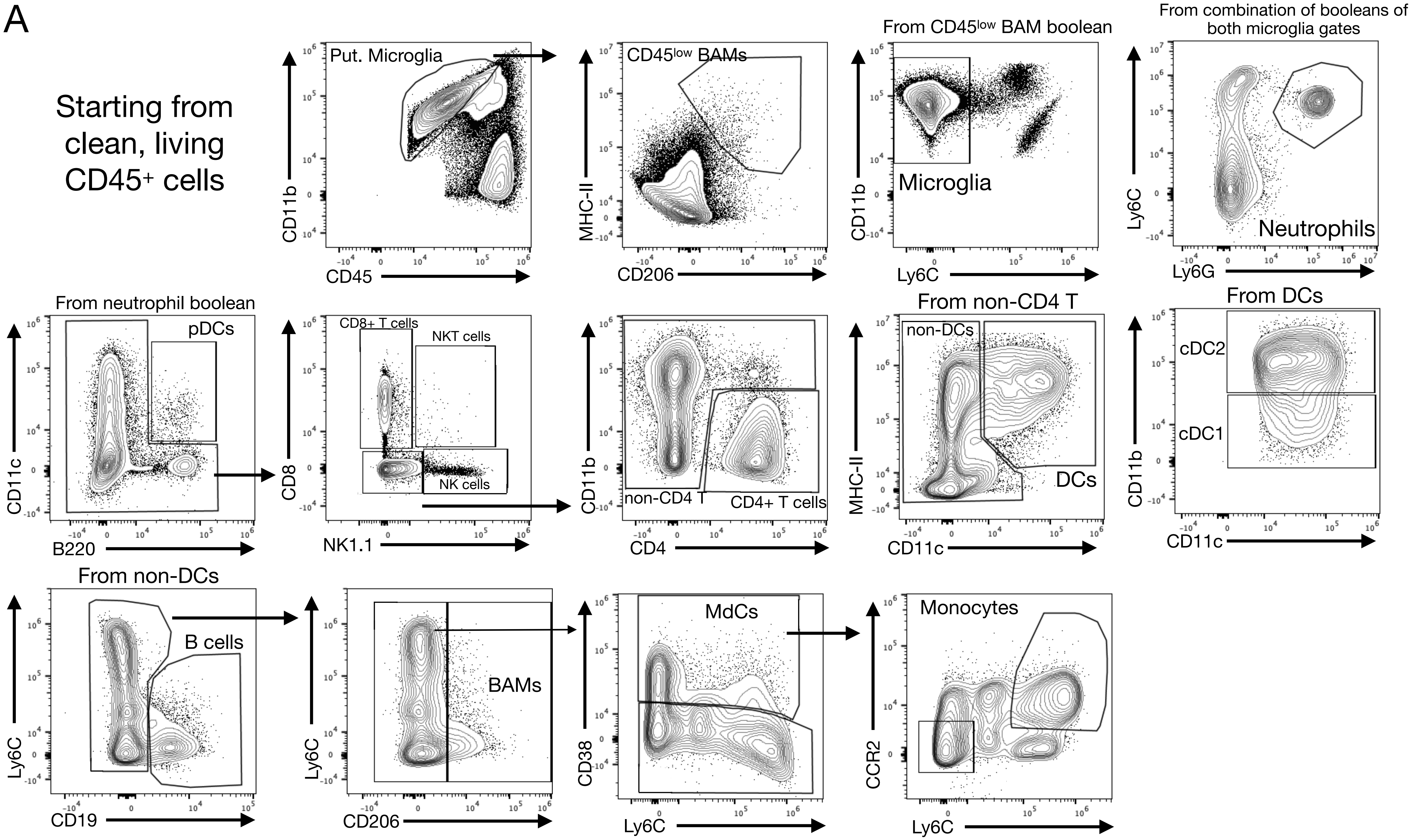

Figure S2. Gating schemes. (A) Gating scheme used to manually annotate nearly all CNS immune cell subsets (used in (Fig. 1H) and (Fig. 2F, G)). This gating scheme is able to identify approximately 98% of all CD45+ CNS immune cells. Put. Microglia, putative microglia; BAM, border-associated macrophage; MdCs, myeloid-derived cells.

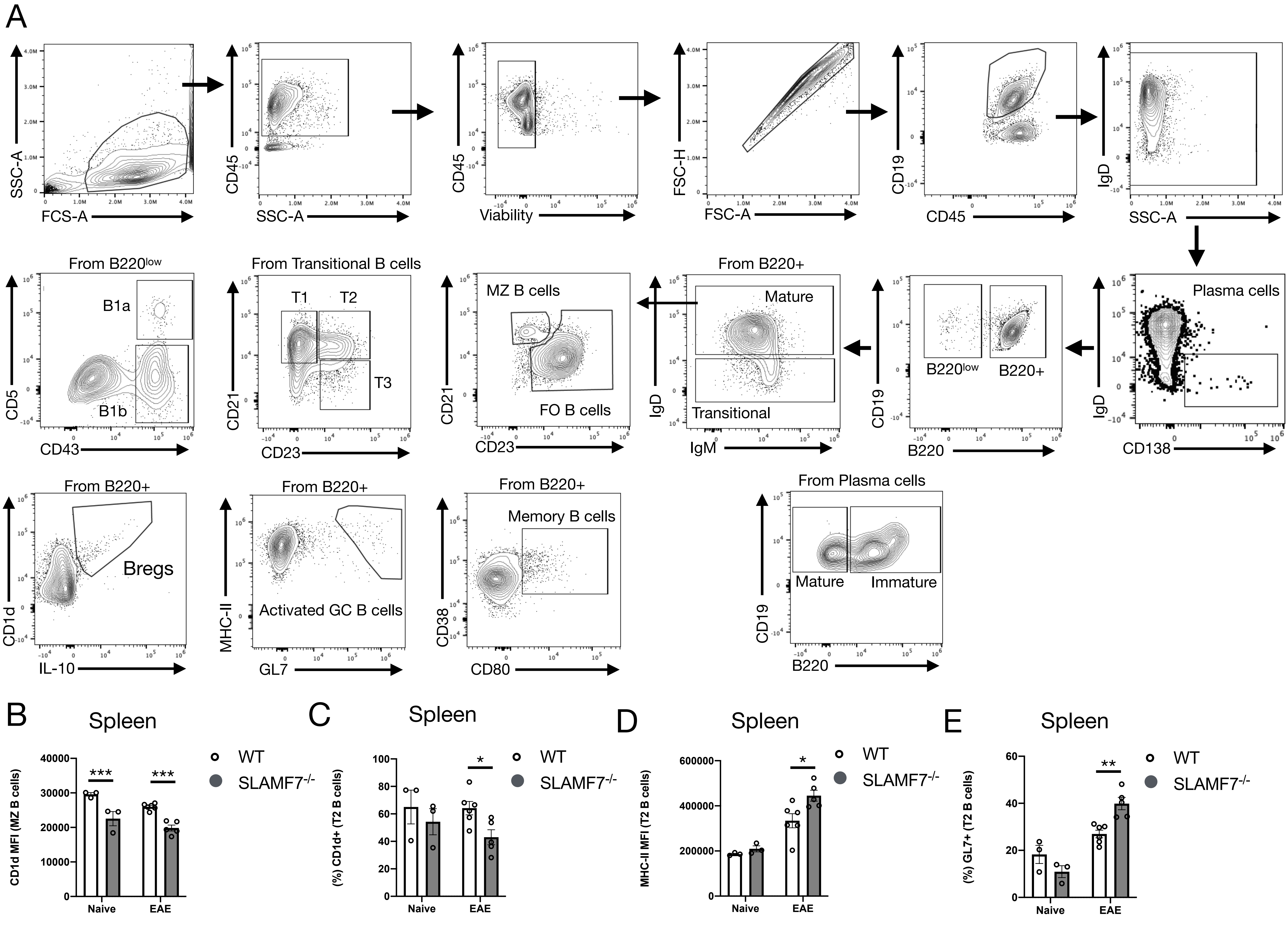

Figure S3. Gating and additional analyses of B cell deep phenotyping (related to Fig. 5). (A) Gating scheme used to clean up datasets and manually annotate all CNS and splenic B cell subsets from high dimensional B cell profiling experiments. (B) CD1d expression on MZ B cells. (C) CD1d expression on T2 B cells. (D) MHC-II expression on T2 B cells. (E) GL7 expression on T2 B cells. Groups in (B-E) compared with a two-way ANOVA with Sidak's test for multiple comparisons. FO, follicular; MZ, marginal zone; Bregs, regulatory B cells. *$p<0.05$, **$p<0.01$, ***$p<0.001$.

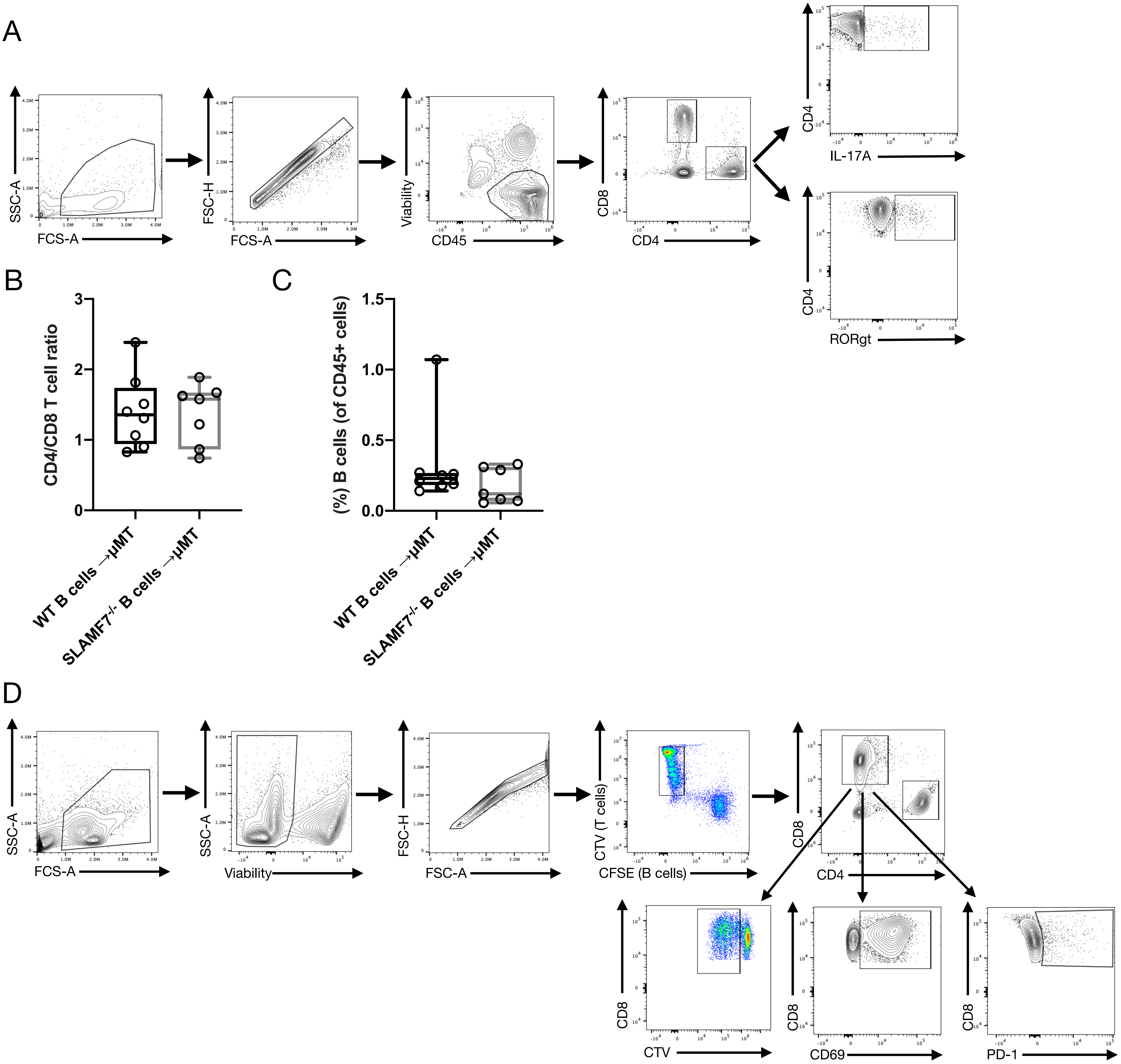

Figure S4. Gating schemes (related to Fig. 6). (A) Gating scheme used to identify IL-17A+ and RORgt+ CD4+ T cells in μMT mice (used in (Fig. 6B, C)). (B) CD4/CD8 T cell ratio in the CNS of mice μMT mice. (C) Frequency of B cells in the CNS of μMT mice. (D) Gating scheme used to measure CD8+ T cell phenotypes from co-culture experiments (used in (Fig. 6L-N)).
