# Supplementary material for: SLAMF7 modulates B cells and adaptive immunity to regulate susceptibility to CNS autoimmunity
Source: J Neuroinflammation. 2022 Oct 3;19:241. doi: 10.1186/s12974-022-02594-9 (PMC9533612; doi:10.1186/s12974-022-02594-9)
Supplement: Supplementary file 2 — Additional file 2: Table S1. Antibody panels used. [file 12974_2022_2594_MOESM2_ESM.docx]

| **Antibody** | **Conjugate** | **Source** |
| --- | --- | --- |
| **Confocal antibodies** | | |
| Anti-goat Iba1 | n/a | Novus |
| Anti-rabbit SLAMF7 (bs-2544R) | n/a | Bioss |
| **Neuroimmune phenotyping panel (IL-10)** | | |
| CD45 (30-F11) | Alexa Fluor 532 | ThermoFischer |
| Cd11b (M1/70) | BV570 | BioLegend |
| Tim3 (B8.2C12) | APC/Fire 750 | BioLegend |
| CD4 (RM4-5) | eFluor 450 | ThermoFischer |
| CD8a (53-6.7) | A700 | eBioscience |
| NK1.1 (PK136) | PE-Cy7 | eBioscience |
| CD19 (1D3) | PerCP-Cy5.5 | Fischer Scientific |
| SLAMF7 (4G2) | APC | R&D Systems |
| NKG2D (CX5) | PE | ThermoFischer |
| Ly-6C (HK1.4) | BV421 | BioLegend |
| Live/Dead | Zombie NIR | BioLegend |
| Ly-6G (1A8) | BV711 | BioLegend |
| MHCII (M5/114.15.2) | BV785 | BioLegend |
| CD3 (17A2) | BUV737 | BD Biosciences |
| Siglec-H (440c) | BV650 | BD Biosciences |
| CD11c (HL3) | PE-CF594 | BD Biosciences |
| B220 (RA3-6B2) | BV480 | Fischer Scientific |
| CD49b (DX5) | PerCP-eFluor 710 | ThermoFischer |
| IgD (IA6-2) | Super Bright 436 | eBiosciene |
| CD206 (C068C2) | Alexa Fluor 647 | BioLegend |
| CD38 (90/CD38) | BV510 | BD Biosciences |
| CCR2 (4575301) | BV750 | BD Biosciences |
| LYVE1 (ALY7) | eFluor 615 | ThermoFischer |
| CD90 (53-2.1) | BUV395 | BD Biosciences |
| Lag-3 (C9B7W) | BUV496 | BD Biosciences |
| **uMT immunophenotyping panel** | | |
| CD45 (30-F11) | Alexa Fluor 488 | BioLegend |
| CD19 (1D3) | PerCP-Cy5.5 | Fischer Scientific |
| CD3 (145-2C11) | APC-Cy7 | BioLegend |
| CD4 (RM4-5) | eFluor450 | ThermoFisher |
| CD8a (53-6.7) | A700 | eBioscience |
| Live/Dead | Zombie NIR | BioLegend |
| T-bet (4B10) | PE Dazzle 594 | BioLegend |
| GATA3 (TWAJ) | eFluor 660 | ThermoFischer |
| IFN gamma (XMG1.2) | APC | eBiosciences |
| TNF alpha (MP6-XT22) | PE-Cy7 | eBiosciences |
| ROR gamma(t) (B2D) | PE | ThermoFischer |
| IL-17 (TC11-18H10.1) | BV785 | BioLegend |
| IgD (IA6-2) | SB436 | ThermoFischer |
| B220 (RA3-6B2) | VioletFluor450 | Fischer Scientific |
| **Neuroimmune phenotyping panel (SF9)** | | |
| CD45 (30-F11) | Alexa Fluor 488 | ThermoFischer |
| Cd11b (M1/70) | BV570 | BioLegend |
| Tim3 (B8.2C12) | APC/Fire 750 | BioLegend |
| CD4 (RM4-5) | eFluor 450 | ThermoFischer |
| CD8a (53-6.7) | A700 | eBioscience |
| NK1.1 (PK136) | PE-Cy7 | eBioscience |
| CD19 (1D3) | PerCP-Cy5.5 | Fischer Scientific |
| CD80 (16-10A1) | PE | eBioscience |
| SLAMF7 (4G2) | APC | R&D Systems |
| Ly-6C (HK1.4) | BV421 | BioLegend |
| Live/Dead | Live/dead Aqua | ThermoFisher |
| Ly-6G (1A8) | BV711 | BioLegend |
| SLAMF9 | Secondary stain w/ Alexa555 | Biorbyt |
| MHCII (M5/114.15.2) | BV785 | BioLegend |
| Siglec-H (440c) | BV650 | BD Biosciences |
| CD11c (HL3) | PE-CF594 | BD Biosciences |
| B220 (RA3-6B2) | BV480 | Fischer Scientific |
| PD-1 (RMP1-30) | PerCP-eFluor 710 | eBiosciences |
| CXCR5 (L138D7) | BV605 | BioLegend |

**Table S1.** Antibody panels used.
